# Interspecific Mating Effects on Locomotor Activity Rhythms and Refractoriness of *Aedes albopictus* (Diptera: Culicidae) Females

**DOI:** 10.3390/insects11120874

**Published:** 2020-12-09

**Authors:** Thais de Souza Feitoza, Victor Henrique Ferreira-de-Lima, Daniel Cardoso Portela Câmara, Nildimar Alves Honório, L. Philip Lounibos, Tamara Nunes Lima-Camara

**Affiliations:** 1 Laboratory of Entomology in Public Health, School of Public Health, University of São Paulo, São Paulo, SP 01246-904, Brazil; thais.byo@live.com (T.d.S.F.); victorhenriquelima274@gmail.com (V.H.F.-d.-L.); 2 Laboratório de Mosquitos Transmissores de Hematozoários, Instituto Oswaldo Cruz, Fundaҫão Oswaldo Cruz, Rio de Janeiro, RJ 21040-360, Brazil; dcpcamara@gmail.com (D.C.P.C.); honorio@ioc.fiocruz.br (N.A.H.); 3 Núcleo Operacional Sentinela de Mosquitos Vetores-Nosmove/Fiocruz, Fundaҫão Oswaldo Cruz, Rio de Janeiro, RJ 21040-360, Brazil; 4 Florida Medical Entomology Laboratory, University of Florida, Vero Beach, FL 32962, USA; lounibos@ufl.edu; 5 Department of Epidemiology, School of Public Health, University of São Paulo, São Paulo, SP 01246-904, Brazil

**Keywords:** *Aedes albopictus*, *Aedes aegypti*, satyrization, locomotor activity, accessory gland

## Abstract

**Simple Summary:**

The superiority of *Aedes albopictus* in larval resource competition was originally proposed as the cause of displacements of *Aedes aegypti* in the USA. However, satyrization, a form of reproductive interference, was later invoked as an alternative or complementary mechanism for the observed displacements of *Aedes aegypti*. This study tests the hypotheses that *Ae. albopictus* female activity is not altered by the presence of accessory gland extracts from conspecific and heterospecific males, and *Ae. albopictus* females remain receptive to conspecific males even after receiving accessory gland (AG) extracts from *Ae. aegypti* males. We performed experiments with a control group (females injected with saline), a group of females injected with accessory gland extracts of *Ae. aegypti* males and a group of females injected with accessory gland extracts of *Ae. albopictus* males and measured the locomotor activity and the ability of inseminated females to copulate with conspecific males. Females injected with conspecific and heterospecific extracts showed significant decreases in total and diurnal activity. Females injected with heterospecific extracts showed significant decreases in nocturnal activity. A total of 83% of females injected with heterospecific and 10% of females injected with conspecific extracts copulated with conspecific males. These results considered together with our previous report on effects of interspecific mating and cross-species injections of AG products on *Ae. aegypti* females, show consistent depressions of locomotor activities between species, but the loss of sexual receptivity only in *Ae. aegypti*. We propose that different male seminal fluid proteins control these activities.

**Abstract:**

This study tests the hypotheses that the locomotor activity of *Ae. albopictus* females is not significantly altered by the presence of accessory gland (AG) extracts from conspecific and heterospecific males, and that *Ae. albopictus* females remain receptive to mating with conspecific males even after receiving AG of *Ae. aegypti* males. Virgin *Ae. albopictus* females were injected with saline (control group), AG extracts of *Ae. aegypti* males (aegMAG) or AG extracts of *Ae. albopictus* males (albMAG). Locomotor activity was evaluated under 12 h of light and 12 h of darkness at 25 °C. All live *Ae. albopictus* females were subsequently exposed to conspecific males for 48 h, and their spermathecae were dissected for the presence of sperm. Females injected with aegMAG and albMAG showed significant decreases in total, diurnal and diurnal without lights-on Period activities. Females injected with aegMAG showed significant decreases in nocturnal and nocturnal without lights-off period activities. Females injected with albMAG showed significant decreases in lights-off activity. A total of 83% of *Ae. albopictus* females injected with aegMAG and 10% of females injected with albMAG were inseminated by conspecific males. These results, coupled with our previous paper on MAG and interspecific mating effects on female *Ae. aegypti*, demonstrate contrasting outcomes on locomotor activities and loss of sexual receptivity, both conspecific and heterospecific MAGs capable of sterilizing virgin *Ae. aegypti*, but only conspecific MAGs sterilizing *Ae. albopictus*, whereas locomotor activities were depressed in females of both species after heterospecific and conspecific injections or treatments.

## 1. Introduction

*Aedes aegypti* (L.) and *Aedes albopictus* (Skuse) are arguably the two most important invasive species of mosquito vectors of public health concern. Although both belong to a common subgenus, *Stegomyia*, and share many behaviors, such as occupancy of containers during immature stages and diurnal feeding and reproductive activities as adults, the two species evolved independently in different regions of the world, and thus, did not meet one another until one or the other’s invasive range expansions led to interspecific encounters [1].

The African origins of *Ae. aegypti* have been long known [2], but only recently has the evolution of the Aegypti Group of species been shown by phylogeographic, molecular research to have originated in islands of the southwestern Indian Ocean [3]. The diaspora of *Ae. aegypti* from continental Africa into the Western Hemisphere in the 15–17th centuries was facilitated by intercontinental shipping and the slave trade [4]. Invasions of this species into Asia came later, as confirmed by historical records of this species invading cities, such as Bangkok and Kuala Lumpur in the early 1800s [5].

The invasions of *Ae. albopictus*, by contrast, have mostly occurred in the 20th century, from its native range in tropical and temperate Asia [4,6]. Earlier establishments of this species in Hawaii and Madagascar were likely progeny of hitchhikers with human travelers to those islands. This species has additionally been able to invade higher, temperate latitudes thanks to an egg diapause, which confers cold-hardiness and is absent in *Ae. aegypti* [7].

Two outcomes, not mutually exclusive, have been reported where *Ae. aegypti* and *Ae. albopictus* populations have met in invasive-invasive or invasive-native ranges: Competitive displacement of *Ae. aegypti* or habitat segregation between the two species [1]. Displacement has only been documented from the SE USA [4] and Bermuda [8]. The competitive displacement of *Ae. aegypti* by *Ae. albopictus* in the SE USA between 1986–1994 was relatively complete, except for large, urban redoubts of *Ae. aegypti*, and persistent through at least two decades in Florida [9]. Yet, no such displacement was observed in Brazil, which was colonized by *Ae. albopictus* about the same time this species became established in the southern USA [10].

Where the two species coexist in sympatry, such as in the southern USA and Brazil, *Ae. aegypti* typically predominates in more urban environments and *Ae. albopictus* in more vegetated habitats, such as suburbs with trees [11]. The superiority of *Ae. albopictus* in larval, resource competition was originally proposed as the cause of competitive displacements of *Ae. aegypti* in the USA [12]. However, after interspecific matings between these two species were discovered to occur in nature in Florida, satyrization, a form of asymmetric mating interference [13], was invoked as an alternative or complementary mechanism for the competitive displacements of *Ae. aegypti* [14]. Although bidirectional matings of these two species were recorded in nature throughout their worldwide ranges in sympatry [15], only the infertile mating of an *Ae. albopictus* male with an *Ae. aegypti* female sterilizes the female recipient, which asymmetry is shown by population models to lead to the possible rapid extinction of the inferior competitor species [16]. The rapid evolution of satyrization resistance in *Ae. aegypti* exposed to male *Ae. albopictus* is believed to facilitate the co-existence of these two species [9,16,17].

As products of the male accessory glands (MAG) of mosquitoes transferred during mating are known to prevent further matings by inseminated females [18], it is not surprising that comparisons of daily activity rhythms showed that virgin females are typically more active than inseminated females in four-vector species [19,20,21,22]. The diminished activities of mated females may be accounted for by the cessation of mate-seeking, and in some species, the diversion of energies to other essential quests, such as blood-feeding.

In the first report of the effects of interspecific matings on mosquito activity rhythms, Lima-Camara et al. [23] described that *Ae. aegypti* females injected with MAG products from *Ae. albopictus* did not differ from saline-injected controls in total locomotor activity during the diel cycle, but did differ in lights-on responses, despite the complete sterilizing effects of the MAGs from *Ae. albopictus* on *Ae. aegypti* females. Recognizing the potentials of such cross-insemination experiments to help decipher the complex roles of MAGs in controlling female behaviors [24], and based on our earlier report that conspecific and heterospecific MAGs injected into *Ae. aegypti* led to decreased locomotor activities [23]; we hypothesize here that analogous injections into virgin *Ae. albopictus* should lead to decreases in locomotor activities of these experimental females. However, based on the previously demonstrated unidirectional sterilizing effect of MAGs in interspecific crosses between these species [13], we hypothesize that virgin *Ae. albopictus* will remain receptive to future conspecific mating after their injection of MAGs from *Ae. aegypti*.

## 2. Materials and Methods

### 2.1. Mosquito Rearing and Dissections of Male Accessory Glands

Both *Ae. aegypti* and *Ae. albopictus* eggs from Rio de Janeiro were obtained from colonies at Laboratório de Mosquitos Transmissores de Hematozoários, Instituto Oswaldo Cruz, Fundação Oswaldo Cruz, state of Rio de Janeiro (RJ), Brazil, as described in Lima-Camara et al. [23]. After eclosion, up to 500 larvae were reared in plastic trays with 1L of tap water and 0.5g of fish food (Tetramin^®^) and were maintained in a room with a photoperiod of light/dark (LD) 12:12, at 25 °C and 70% relative humidity. Pupae of both species were individually placed in small glass containers with 5 mL of tap water to ensure virgin males and females after emergence [23].

Twenty-five 5–7 day-old virgin male *Ae. aegypti* and *Ae. albopictus* were dissected under a stereoscopic microscope to remove their accessory glands (Ags) [25]. Twenty-five pairs of Ags of each species were stored in 50 µL of saline, in a proportion of 1 AG to each 1 µL of saline, at −20 °C. Prior to injections, the AG solutions of both species were sonicated for 1 min and centrifuged for 1 h at 13,000 *g* [23].

### 2.2. Injections of Ae. albopictus Females and Analysis of Locomotor Activity

Six to seven day-old virgin *Ae. albopictus* females were separated into three groups of 64 females each. Females from the first group (control group) were individually injected by intrathoracic inoculation with 0.28 µL of saline; whereas, the second and third groups (MAG groups) were intrathoracically inoculated with 0.28 µL of male accessory gland extract (MAG) solution of *Ae. aegypti* and 0.28 µL of MAG solution of *Ae. albopictus*, respectively, using a Nanoject microinjector (Drummond Scientific). Non-injected virgin females of *Ae. albopictus* were not included as controls in the present study. Results of locomotor activity of virgin and naturally inseminated females of *Ae. albopictus* were published previously [22]. The volume of 0.28 µL corresponds to more than a quarter of a MAG, which is sufficient for females to respond as if they have been conspecifically inseminated [18,23]. We have opted for the intrathoracic injections because interspecific insemination under laboratory conditions is extremely low [23,26], which would potentially impact the number of inseminated females required for the locomotor activity experiments.

Each injected *Ae. albopictus* female (control and both MAG groups) was individually placed in a cylindrical glass tube (1 cm × 10 cm) with a cotton plug soaked in 10% sucrose at one of the extremities for feeding. The glass tubes were sealed with Parafilm^®^ and then placed in a larger version of the Drosophila Activity Monitor (TriKinetics Inc, Waltham, MA, USA) as described in Lima-Camara et al. [22,23]. All monitors were placed in a Precision Scientific Model 818 Incubator, under a constant temperature of 25 °C and a photoperiod of 12 h of light and 12 h of dark (LD 12:12). For each *Ae. albopictus* female, the total locomotor activity during 30-min intervals was continuously recorded for six consecutive days after inoculation, using the DAM System Data Collection program (TriKinetics Inc., Waltham, MA, USA). Two replicates were conducted, and only control and MAG-injected females that lived until the fourth day of the locomotor activity recordings (five days after inoculation) were considered for analysis.

At the end of the locomotor activity experiment, all live *Ae. albopictus* females of each group were counted and transferred to three different cages (17 cm × 17 cm) containing conspecific virgin males in a ratio of 1 female: 2 males. After 48 h, all three spermathecae were dissected from live females for detection of *Ae. albopictus* sperm under a compound microscope at 100 × magnification.

### 2.3. Statistical Analysis

For statistical analysis, we transformed the locomotor activity values of all mosquitoes to log (N + 1), in order to avoid the influence of very high or very low values frequently observed in the activity of mosquitoes and to accommodate the zero values observed in the data series [27]. We calculated the modified geometric William’s mean (M_w_) as an estimate of the central tendency of activity during each time interval for exploratory analysis and figures [22,23,28,29]. To compare the locomotor activity between females of *Ae. albopictus* injected with saline *versus* females of *Ae. albopictus* injected either with *Ae. aegypti* male AG (aegMAG) or *Ae. albopictus* male AG (albMAG), we calculated seven indices [22,23]: (i) total activity, (ii) diurnal activity, (iii) diurnal activity without lights-on period, i.e., the activity during the photophase except for the first 30 min, which corresponds to the morning activity peak, (iv) lights-on activity, which corresponds to the first 30 min just after lights-on, (v) nocturnal activity, (vi) nocturnal activity without lights-off period, i.e., the activity during the scotophase except for the first 30 min, which corresponds to the evening activity peak, and (vii) lights-off activity, which corresponds to the first 30 min just after lights-off.

We were interested in testing the effect of the different accessory gland origins on female *Ae. albopictus* locomotor activity. Because we have repeated measures for each individual mosquito across four days of continuous experiments, we opted to use generalized linear mixed models (GLMM) with a Gaussian distribution [30], using the lme4 package in R [31]. We created one different model for each of the seven indices mentioned above. All models included a random effect for mosquito ID to account for the repeated measurements. As fixed effects in all models, we were mainly interested in “status” (a categorical variable with three levels: Control, as the baseline level; *Ae. aegypti* MAG; and *Ae. albopictus* MAG). We also included “block” (a dichotomous variable for experiments 1 and 2) and a two-way interaction between status and block. Assumptions of homoscedasticity and normality of residuals were checked via graphical evaluation [32]. F-tests to test if the inclusion of the two fixed effects and the interaction term was significant, and Tukey post-hoc analyses were performed using the lmerTest package [33]. All analyses were done using R (4.0.1) [34] and Rstudio (1.2.5033) [35].

## 3. Results

**Figure 1:**
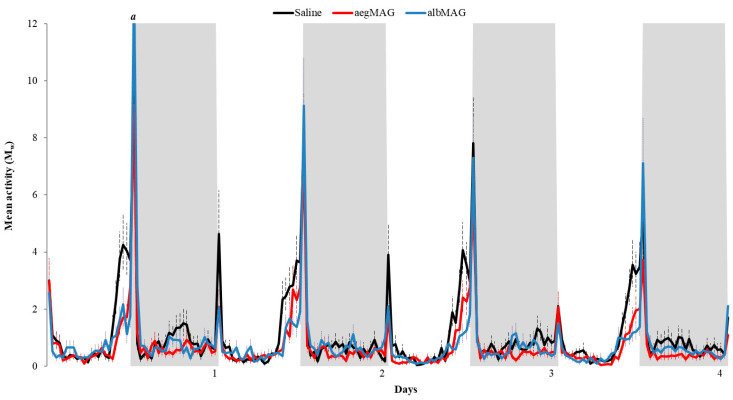
William’s mean (M_w_) of locomotor activity of *Aedes albopictus* virgin females injected with saline (black line-control group; n = 63), male accessory gland (MAG) of *Ae. aegypti* (red line; n = 70) and MAG of *Aedes albopictus* (blue line; n = 74) exposed to four days of 12 h of light (white columns) and 12 h of dark (grey columns) (light/dark 12:12) at 25 °C. Dotted bars represent 1 standard error of the M_w_. A reading exceeding the scale is indicated with the letter *a* and its value is *a* = 13.48 (+1.92).

**Figure 2:**
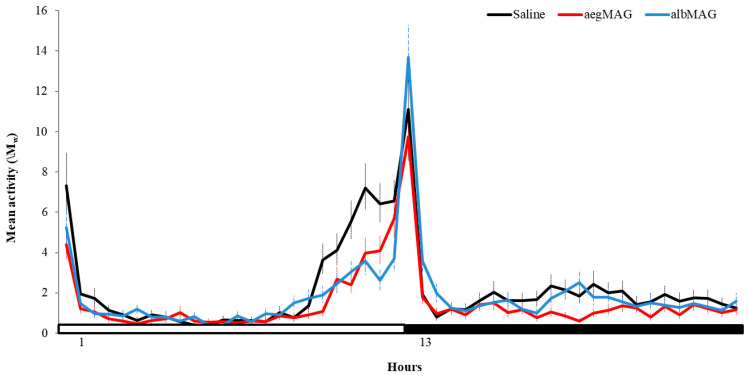
William’s mean (M_w_) of locomotor activity of *Aedes albopictus* virgin females injected with saline (black line-control group; n = 63), MAG of *Ae. aegypti* (red line; n = 70) and MAG of *Aedes albopictus* (blue line; n = 74) under 12 h of light (white bar) and 12 h of dark (black bar) (light/dark 12:12) at 25 °C. Lines represent the 30 min mean activity (±standard error) of control and MAG-injected females in the four tested days.

In both control and MAG injection groups, *Ae. albopictus* females showed a bimodal rhythm, with peaks at lights-on and lights-off (Figure 1 and Figure 2). Overall, the control group showed higher lights-on peaks, whereas *Ae. albopictus* females injected with MAG substance from *Ae. albopictus* showed the highest peak at lights-off (Figure 1 and Figure 2). *Aedes albopictus* females injected with MAGs showed a general decrease in locomotor activity in all the four tested days compared to control females (Figure 1). This becomes more evident in the graph of the average of the four days, which indicates the lower locomotor activity in both MAG injected groups, especially at the end of the photophase (Figure 2).

F-tests for the GLMM models indicated that the inclusion of status was significant for total activity (F_2, 201_ = 4.3296, *p* < 0.05); diurnal activity (F_2, 201_ = 5.1346, *p* < 0.01); diurnal activity without lights-on (F_2, 201_ = 4.5596, *p* < 0.05); and lights-on activity (F_2, 201_ = 4.6340, *p* < 0.05). We found borderline significance for nocturnal activity (F_2, 201_ = 2.9092, *p* = 0.05682). Status was not significant for both nocturnal activity without lights-off (F_2, 201_ = 2.7769, *p* = 0.06462) and lights-off activity (F_2, 201_ = 2.5070, *p* = 0.08406) (Table 1).

F-tests results also indicated no significant block effects, except for the lights-on mean (F_1, 201_ = 6.9824; *p* < 0.01). However, in both blocks, the lights-on peak of the control group was higher (0.59 and 0.74, for block 1 and 2, respectively) than the females injected with conspecific MAG (0.33 and 0.57 for block 1 and 2, respectively) and heterospecific MAG (0.48 and 0.54 for block 1 and 2, respectively). No significant interactions between block and status were observed in any of GLMMs (*p* > 0.05) (Table 2).

The total locomotor activity of females injected with *Ae. aegypti* and with *Ae. albopictus* MAG was significantly lower (mean ± SE; 0.199 ± 0.004 and 0.222 ± 0.004, respectively) than the control group (0.264 ± 0.004). The same pattern of higher locomotor activity in the control group was observed for the diurnal activity (0.270 ± (0.007) vs. 0.199 ± (0.006) and 0.201 ± (0.006), respectively for control, aegMAG and albMAG), diurnal activity without lights-on (0.254 ± (0.007) vs. 0.186 ± (0.006) and 0.189 ± (0.006)) and lights-on activity (0.634 ± (0.043) vs. 0.503 ± (0.036) and 0.483 ± (0.037)) (Table 1). No status effect was found in the F-tests for nocturnal activity, nocturnal activity without lights-off, and lights-off activity (Table 1 and Table 2).

**Figure 3:**
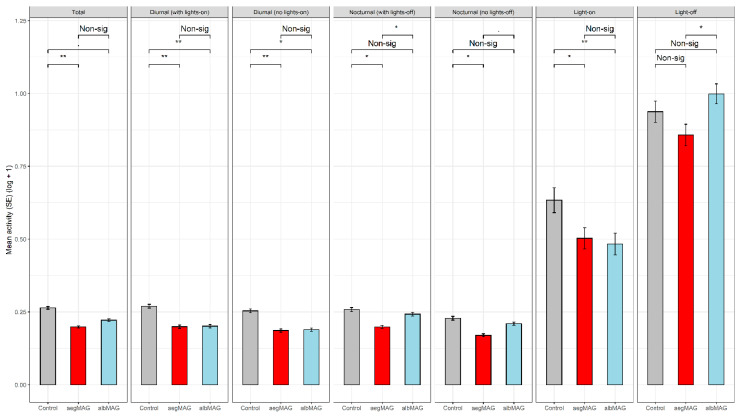
Total activity, diurnal activity, diurnal activity without lights-on, nocturnal activity, nocturnal activity without lights-off, lights-on activity and lights-off activity of *Ae. albopictus* females injected with saline (Control; n = 63), accessory gland of *Ae. aegypti* males (aegMAG; n = 70) and accessory gland of *Ae. albopictus* males (albMAG; n = 74). * = significant at alpha = 0.05; ** significant at alpha = 0.001; Non-sig = Non-significant at alpha = 0.05.

Model results showed that when controlling for all other variables, females of *Ae. albopictus* injected with aegMAG and albMAG showed a significant decrease in the total activity group in relation to control females (*p* < 0.01 and *p* < 0.05, respectively). Both MAG injected females also showed significant decreases in the diurnal activity (aegMAG, *p* < 0.01; albMAG, *p* < 0.01) and in the diurnal activity without lights-on (aegMAG, *p* < 0.01; albMAG, *p* < 0.05) groups in relation to control females (Figure 3). Regarding nocturnal activity group, a significant decrease was reported for *Ae. albopictus* females injected with aegMAG in relation to control females (*p* < 0.05), even when we did not consider the lights-off in the nocturnal activity without lights-off group (*p* < 0.05). For the lights-on activity group, a significant decrease was observed just in *Ae. albopictus* females injected with albMAG (*p* < 0.01), and no significant difference was reported for the lights-off activity group (*p* > 0.05) (Figure 3).

Table 3 shows the analysis of the spermathecae of all live *Ae. albopictus* females that were exposed to conspecific males for 48 h at the end of the locomotor activity experiments. Most females of *Ae. albopictus* injected with saline had positive spermathecae (87%). A similar proportion of positive spermathecae was observed in females injected with heterospecific MAG (83%), that is, females of *Ae. albopictus* copulated with their conspecific males, even though they were injected with AG from *Ae. aegypti* males. However, only 10% of *Ae. albopictus* females injected with conspecific MAG had positive spermathecae (Table 3).

## 4. Discussion

Our results confirm Tripet et al. [14], who showed that MAG products from *Ae. aegypti* do not render virgin *Ae. albopictus* refractory to subsequent matings, thus supporting the conclusion of the asymmetry of reproductive interference between these two species, favoring *Ae. albopictus*. The interspecific effects of MAGs from *Ae. aegypti* causing decreases in locomotor activity rhythms of *Ae. albopictus* are consistent with previous results showing decreased activities of *Ae. aegypti* following injections of *Ae. albopictus* MAGs [23]. Coupling the similarities in locomotor responses of the two species with the different effects on their reproductive behaviors, one may infer that the seminal fluid proteins (Sfps) from MAGs that influence female reproductive behaviors may differ from those that govern locomotory behaviors. In order to link Sfps with specific behaviors, activity rhythms simply recorded as movements would need to be decomposed into recognizable fixed action patterns. Such decomposition of the activity recordings has been accomplished by Jones [20] for *Ae. aegypti*, by Lima-Camara et al. for *Ae. aegypti* and *Ae. albopictus* [22] and Jones and Gubbins [21] for *Culex pipiens quinquefasciatus* by comparing recordings during the day/night cycles of females in different physiological states, such as virgin/inseminated, blood-fed/sugar-fed, and parous/nulliparous.

Although the strength of evidence varies among phenomena, Klowden [24] listed the following female behaviors as being controlled by MAGs, passed to females during mating as Sfps: Inhibition of mating; stimulation of oviposition; switch to pre-oviposition behavior; circadian rhythmicity; and modulation of host-seeking. He further observed, as confirmed by the unidirectional effect on mating inhibition in the interspecific cross between *Ae. aegypti* and *Ae. albopictus*, that activation of some behaviors and not others, depends on the donor accessory gland, indicating that some components may be present in the males of a few species, but are absent in males of other species or inactive in some females. Multiple components may be needed to activate behaviors in some species, and some Sfps may control multiple behaviors, such as mating inhibition and oviposition. Heterologous transfers of MAGs between species, as performed in this report and by Lima-Camara et al. [23], may help to resolve the species-specific, Sfps-behavior relationships.

Degner et al. [36] identified 280 Sfps from male *Ae. aegypti* and Boes et al. [37] recognized 198 Sfps in male *Ae. albopictus*. Although Boes et al. [36] did not have access to the more complete sperm proteome of Degner et al. [36], using an earlier estimate of Sfps in the yellow fever mosquito, only 36.4% of the Sfps in *Ae. albopictus* were regarded as orthologs of Sfps in *Ae. aegypti* [37], confirming the evolutionary divergence between these two *Stegomyia* spp. Based on functional categories, only 0.5% of the Sfps from *Ae. albopictus* were classified as hormones [37], but the Sfps of both species were rich in proteins that regulate proteolysis [36,37], suggesting that catalytic reactions stimulated by Sfps could activate releases of behaviors in the mated female.

As observed by Klowden [24,38] and Gillott [39], specific proteins produced by MAGs and transferred to females during mating can influence reproductive and feeding behaviors of inseminated *Aedes* females, among other behavioral effects. Lima-Camara et al. [22] evaluated the locomotor activity of inseminated *Ae. albopictus* females after releasing virgin *Ae. albopictus* females inside a cage with approximately 100 conspecific males for 24 h. The authors reported a non-significant decrease in all of the parameters evaluated for a locomotor activity for inseminated *Ae. albopictus* females compared to virgin *Ae. albopictus* females, including total, diurnal and diurnal without lights-on activities [22]. These results differ from those observed in the present study, which may be related to the way in which these females had contact with the AG compounds of the males, either by direct contact with males during mating and/or male harassment. In the study of Lima-Camara et al. [22], the females of *Ae. albopictus* were naturally inseminated by their conspecific males, whereas in the present study, we injected the equivalent of more than a quarter (0.28) of an AG into each *Ae. albopictus* female. This dose is more than sufficient for females to respond as if they have been inseminated, since each male apparently could sterilize at least 64 females [18].

Previous laboratory experiments have indicated that *Ae. aegypti* females are more likely to be inseminated interspecifically than *Ae. albopictus* females, supporting the competitive advantage of the former species [26,40,41]. Nazni et al. [41] reported that *Ae. aegypti* females inseminated by *Ae. albopictus* males produced more eggs than *Ae. albopictus* females inseminated by *Ae. aegypti* males, although no eggs were viable. Moreover, Bargielowski et al. [26] observed that cross-inseminations between *Ae. albopictus* females and *Ae. aegypti* males in cages are significantly less common than between *Ae. aegypti* females and *Ae. albopictus* males. In nature, the frequencies of cross-insemination between *Ae. aegypti* and *Ae. albopictus* were estimated in collections from different countries, such as the United States [14,15], Venezuela, Gabon, and Singapore [15]. Despite the low rates of cross-mating that were observed (1.12–3.73%), the results indicated that *Ae. aegypti* females were more likely to be inseminated interspecifically than *Ae. albopictus* females under uncontrolled conditions in the field [14,15]. Moreover, frequencies of satyrization based on the presence or absence of interspecific sperm in spermathecae may be underestimated. A laboratory study demonstrated that satyrization of *Ae. aegypti* by *Ae. albopictus* may occur without evidence of successful insemination [42]. Thus, *Ae. aegypti* females exposed to *Ae. albopictus* males may receive MAGs that inhibit subsequent conspecific mating even though their spermathecae contain no heterospecific sperm [42]. Honório et al. [10] conducted a series of laboratory experiments on cross-insemination using *Ae. aegypti* females and *Ae. albopictus* males from several Brazilian and North American cities. These authors showed that *Ae. albopictus* male origin might be a key driver of their satyrization potential upon *Ae. aegypti* females, which might explain why the displacement of the former species was pronounced in North America, but undetected in Brazil [10]. These findings support the need for further investigations on the effect of MAGs of males of *Ae. aegypti* and *Ae. albopictus* from different origins on the behaviors and activities of inseminated females. As the insemination status of a female (either by a conspecific or heterospecific male) is one of the key drivers of satyrization, our results show that this ecological interaction might have very complex effects on the life history of both species. Because of a higher proportion of *Ae. albopictus* females injected with heterospecific and a lower proportion of females injected with conspecific extracts copulated with conspecific males, satyrization plays an important role that shapes the distribution of *Ae. aegypti* and *Ae. albopictus* [26,40,41]. Moreover, the statistically significant impact of conspecific and heterospecific MAG extracts on the locomotor activities of *Ae. albopictus* females (the former impacting fewer activity indexes than the later) shows that important biological traits and behaviors might be modulated in a very complex manner and deserve further studies [24]. Some of these traits, such as host-seeking modulation would benefit from further studies where an experimental design that would test for host-cues under controlled conditions, such as CO_2_ and chemical compounds, to examine if the diurnal locomotor activity would be impacted by the presence of conspecific and/or heterospecific MAGs. Furthermore, other experimental conditions could be manipulated to produce further results, such as using different lighting conditions, photoperiods, temperature and/or humidity, etc.

## 5. Conclusions

Understanding how MAGs control female behaviors are important for vector physiology/molecular biology, because of their potential for contributing to vector and disease control. The fortuitous interspecific encounters of *Ae. aegypti* and *Ae. albopictus* brought about by mosquito invasions, and their capacities to cross-inseminate, could play a role in helping to answer these questions. For the first time, we demonstrated that both MAGs of *Ae. aegypti* and *Ae. albopictus* decrease the locomotor activity of *Ae. albopictus* females. Moreover, this study confirms that MAG of *Ae. aegypti* does not make *Ae. albopictus* females refractory to mating with conspecific males. Thus, we believe that further studies to investigate the effects of cross-insemination, would illuminate finer details of reproductive interference between these species, as well as the control of female behaviors by male accessory gland products.

## Figures and Tables

**Table 1:** Locomotor activity indices (standard error) of *Ae. albopictus* females injected with saline, MAG of *Ae. aegypti* and MAG of *Ae. albopictus*.

	Saline (n = 63)	*Ae. aegypti* MAG (aegMAG) (n = 70)	*Ae. albopictus* MAG (albMAG) (n = 74)	F Test *^b^*
Total Activity	0.264 (0.005)	0.199 (0.004)	0.222 (0.004)	F_2, 201_ = 4.3296, *p* < 0.05
Diurnal Activity	0.270 (0.007)	0.199 (0.006)	0.201 (0.006)	F_2, 201_ = 5.1346, *p* < 0.01
Diurnal Activity Without Lights-on	0.254 (0.007)	0.186 (0.006)	0.189 (0.006)	F_2, 201_ = 4.5596, *p* < 0.05
Lights-on Activity	0.634 (0.043)	0.503 (0.036)	0.483 (0.037)	F_2, 201_ = 4.6340, *p* < 0.05
Nocturnal Activity	0.258 (0.007)	0.198 (0.006)	0.242 (0.006)	F_2, 201_ = 2.9092, *p* = 0.05682
Nocturnal Activity Without Lights-off	0.228 (0.006)	0.170 (0.005)	0.209 (0.006)	F_2, 201_ = 2.7769, *p* = 0.06462
Lights-off Activity	0.937 (0.036)	0.857 (0.037)	0.999 (0.034)	F_2, 201_ = 2.5070, *p* =0.08406

Transformed log + 1 values were used for calculating activities and for statistics. *b*: Result of F-test to include the “status” fixed effect in the GLMM; df, degrees of freedom.

**Table 2:** Estimated fixed effects of status, block, and status x block interaction in *Ae. albopictus* females. Bold entries indicate statistically significant results (*p* < 0.05).

**Fixed Effects**	**Diurnal Activity**				
	**Estimate**	**SE**	**DF**	**T Value**	***p*** **-Value**
Intercept	**0.27893**	**0.0171**	**201**	**16.314**	**<0.001**
Block: Experiment 2	−0.0342	0.03291	201	−1.039	0.30003
Status: *Ae. aegypti* MAG	**−0.07935**	**0.02507**	**201**	**−3.165**	**<0.01**
Status: *Ae. albopictus* MAG	**−0.07572**	**0.0278**	**201**	**−2.724**	**<0.01**
Block: Experiment 2 x Status: *Ae. aegypti* MAG	0.03362	0.04322	201	0.778	0.43748
Block: Experiment 2 x Status: *Ae. albopictus* MAG	0.03069	0.04308	201	0.712	0.47712
**Fixed effects**	**Diurnal Activity Without Lights-on**				
	**Estimate**	**SE**	**DF**	**T Value**	***p*** **-Value**
Intercept	**0.26529**	**0.01656**	**201**	**16.022**	**<0.001**
Block: Experiment 2	−0.0423	0.03188	201	−1.327	0.18603
Status: *Ae. aegypti* MAG	**−0.07771**	**0.02428**	**201**	**−3.201**	**<0.01**
Status: *Ae. albopictus* MAG	**−0.0678**	**0.02692**	**201**	**−2.519**	**<0.05**
Block: Experiment 2 x Status: *Ae. aegypti* MAG	0.03894	0.04185	201	0.93	0.35326
Block: Experiment 2 x Status: *Ae. albopictus* MAG	0.02827	0.04172	201	0.678	0.49886
**Fixed effects**	**Lights-on Activity**				
	**Estimate**	**SE**	**DF**	**T Value**	***p*** **-Value**
Intercept	**0.59269**	**0.05714**	**201**	**10.373**	**<0.001**
Block: Experiment 2	0.15206	0.11	201	1.382	0.16837
Status: *Ae. aegypti* MAG	−0.11711	0.08378	201	−1.398	0.16373
Status: *Ae. albopictus* MAG	**−0.25776**	**0.09289**	**201**	**−2.775**	**<0.01**
Block: Experiment 2 x Status: *Ae. aegypti* MAG	−0.08873	0.14443	201	−0.614	0.53967
Block: Experiment 2 x Status: *Ae. albopictus* MAG	0.08631	0.14397	201	0.599	0.54954
**Fixed effects**	**Nocturnal Activity**				
	**Estimate**	**SE**	**DF**	**T Value**	***p*** **-Value**
Intercept	**0.27335**	**0.01913**	**201**	**14.293**	**<0.001**
Block: Experiment 2	−0.05689	0.03682	201	−1.545	0.1239
Status: *Ae. aegypti* MAG	**−0.06134**	**0.02804**	**201**	**−2.187**	**<0.05**
Status: *Ae. albopictus* MAG	−0.03111	0.03109	201	−1.001	0.3182
Block: Experiment 2 x Status: *Ae. aegypti* MAG	0.02493	0.04834	201	0.516	0.6067
Block: Experiment 2 x Status: *Ae. albopictus* MAG	0.05709	0.04819	201	1.185	0.2376
**Fixed effects**	**Nocturnal Activity Without Lights-off**				
	**Estimate**	**SE**	**DF**	**T Value**	***p*** **-Value**
Intercept	**0.24321**	**0.0184**	**201**	**13.215**	**<0.001**
Block: Experiment 2	−0.05467	0.03543	201	−1.543	0.1244
Status: *Ae. aegypti* MAG	**−0.06043**	**0.02699**	**201**	**−2.239**	**<0.05**
Status: *Ae. albopictus* MAG	−0.03788	0.02992	201	−1.266	0.2069
Block: Experiment 2 x Status: *Ae. aegypti* MAG	0.02406	0.04652	201	0.517	0.6055
Block: Experiment 2 x Status: *Ae. albopictus* MAG	0.06136	0.04637	201	1.323	0.1873
**Fixed effects**	**Lights-off Activity**				
	**Estimate**	**SE**	**DF**	**T Value**	***p*** **-Value**
Intercept	**0.96659**	**0.06406**	**201**	**15.09**	**<0.001**
Block: Experiment 2	−0.10789	0.12331	201	−0.875	0.383
Status: *Ae. aegypti* MAG	−0.08222	0.09392	201	−0.875	0.382
Status: *Ae. albopictus* MAG	0.1246	0.10414	201	1.197	0.233
Block: Experiment 2 x Status: *Ae. aegypti* MAG	0.04482	0.16191	201	0.277	0.782
Block: Experiment 2 x Status: *Ae. albopictus* MAG	−0.04112	0.1614	201	−0.255	0.799
**Fixed effects**	**Total Activity**				
	**Estimate**	**SE**	**DF**	**T Value**	***p*** **-Value**
Intercept	**0.27614**	**0.01498**	**201**	**18.432**	**<0.001**
Block: Experiment 2	−0.04554	0.02884	201	−1.579	0.11587
Status: *Ae. aegypti* MAG	**−0.07035**	**0.02197**	**201**	**−3.202**	**<0.01**
Status: *Ae. albopictus* MAG	**−0.05341**	**0.02436**	**201**	**−2.193**	**<0.05**
Block: Experiment 2 x Status: *Ae. aegypti* MAG	0.02928	0.03787	201	0.773	0.44038
Block: Experiment 2 x Status: *Ae. albopictus* MAG	0.04389	0.03775	201	1.163	0.24638

**Table 3:** Number and percentage of positive and negative dissected spermathecae of *Aedes albopictus* females injected with saline (control), accessory glands MAGs of *Ae. aegypti* and MAGs of *Aedes albopictus* and exposed to conspecific males for 48 h.

Injection	Positive Spermathecae n (%)	Negative Spermathecae n (%)
Saline (Control)	33 (86.84%)	5 (13.16%)
*Ae. aegypti* MAG (aegMAG)	25 (83.33%)	5 (16.67%)
*Ae. albopictus* MAG (albMAG)	2 (10.00%)	18 (90.00%)
Total	60 (68.18%)	28 (31.82%)
